# Computational Methods for Detection of Differentially Methylated Regions Using Kernel Distance and Scan Statistics

**DOI:** 10.3390/genes10040298

**Published:** 2019-04-12

**Authors:** Faith Dunbar, Hongyan Xu, Duchwan Ryu, Santu Ghosh, Huidong Shi, Varghese George

**Affiliations:** 1 Genome Research Center, AbbVie, North Chicago, IL 60064, USA; fengjiao.dunbar@abbvie.com; 2 Department of Population Health Sciences, Augusta University, Augusta, GA 30912, USA; hxu@augusta.edu (H.X.); sghosh@augusta.edu (S.G.); 3 Division of Statistics, Northern Illinois University, DeKalb, IL 60115, USA; dryu@niu.edu; 4 Georgia Cancer Center, Augusta University, Augusta, GA 30912, USA; hshi@augusta.edu

**Keywords:** binomial scan statistic, CpG sites, DNA methylation, kernel distance statistic, mixed-effects model

## Abstract

Motivation: Researchers in genomics are increasingly interested in epigenetic factors such as DNA methylation because they play an important role in regulating gene expression without changes in the sequence of DNA. Abnormal DNA methylation is associated with many human diseases. Results: We propose two different approaches to test for differentially methylated regions (DMRs) associated with complex traits, while accounting for correlations among CpG sites in the DMRs. The first approach is a nonparametric method using a kernel distance statistic and the second one is a likelihood-based method using a binomial spatial scan statistic. The kernel distance method uses the kernel function, while the binomial scan statistic approach uses a mixed-effects model to incorporate correlations among CpG sites. Extensive simulations show that both approaches have excellent control of type I error, and both have reasonable statistical power. The binomial scan statistic approach appears to have higher power, while the kernel distance method is computationally faster. The proposed methods are demonstrated using data from a chronic lymphocytic leukemia (CLL) study.

## 1. Introduction

Genetic variations from genome-wide association studies can explain only a small proportion of the phenotypic variation for most diseases [1]. It has been established that most diseases are caused by both genetic factors and non-genetic factors such as environmental factors, contributing to epigenetic changes, especially changes in DNA methylation at CpG sites. For example, research has found that aberrant DNA methylation of multiple promoter-associated CpG islands can suppress gene expression by inactivating the function of tumor suppressor genes, eventually causing cancer [2].

Methylation data from next-generation sequencing (NGS) such as Methyl-seq have been used to detect aberrant DNA methylation [3]. NGS coupled with bisulphite treatment of DNA converts unmethylated cytosines to uracils and leaves methylated cytosines intact. This results in counts of uracils (unmethylated) and cytosines (methylated) at each CpG site for every sample. The total counts of uracils and cytosines are the sequencing coverage at each CpG site, which could be different for each sample. Samples with large sequencing coverage could have undue influence in statistical analysis. In order to avoid that, the methylation rate at each site has been suggested for analysis, which is the ratio of methylated alleles over the sequencing coverage at each site. 

Methylation rates are treated as continuous when measured across a large number of cells [4]. The rates at nearby CpG site have been shown to be correlated with a complicated structure [5]. Recent research focus has expanded to incorporate patterns of methylation in clusters of CpG sites, referred to as differentially methylated regions (DMRs) in the genome. 

Many statistical methods have been developed to detect DMRs, including some general approaches for bump detection, such as bump-hunting techniques [6]. Other methods, such as BSmooth [7] and BiSeq [8] are developed specifically for detecting DMRs based on bisulfite sequencing data. Both these methods use functional data analysis methods, where the functional relationship between methylation and location is modeled to estimate a subject-specific profile. 

BSmooth tests the group differences via a test that is similar to a *t*-test at each CpG site. DMRs are defined as adjacent CpG sites with observed values of the *t*-statistic above a pre-defined threshold, and with the significance of the DMRs evaluated using permutation test. However, this method depends on the pre-defined threshold for the *t*-statistic, which would hinder automated analysis and, possibly, lead to biased conclusions. 

In order to make improvements, BiSeq uses a False Discovery Rate (FDR) procedure to control the expected proportion of incorrectly rejected regions. BiSeq also has the advantage of taking spatial dependence into account. Besides that, BiSeq can improve power with a hierarchical procedure in which it starts with a beta-binomial model to account for biological variation between replicates, and then tests significance at each CpG site in all target regions for methylation differences, with a triangular kernel to capture the step-like changes observed in their data. The resulting *p*-values for the CpG sites are transformed into normalized *z*-scores, and then the average is calculated for a given region, and compared to those obtained from resampling data. 

Ryu et al. [9] suggested using wavelets for data smoothing in the functional data analysis for DMRs. Their generalized integrated function test (GIFT) estimates subject-specific functional profiles first by using wavelets, and averaging profiles within groups. An ANOVA-like test is used for testing group differences for a region, by comparing the overall functional relationship to the average curve within each group. This method mainly focuses on testing for differential methylation of a region, which needs other tools to define candidate regions first. 

It has been shown that methylation rates could be strongly associated with relevant predictors and other covariates such as age [10,11] and gender [12,13]. Therefore, in addition to properly accounting for the within and between CpG sites dependence, it is also important to adjust for these covariates in the model, especially for methylation data, since it could bias effect size estimate. 

In this paper, we propose two methods for DMR detections, one based on a kernel distance statistic (KDM) and the other based on a binomial scan statistic method (SSM). A kernel distance statistic, $Q=r^{\prime }Ar,$ where r is a vector of relative frequencies and **A** is a pre-defined matrix of a measure of closeness between two points, was first introduced by Tango [14], to detect geographical clustering of disease. **A** is referred to as the kernel matrix by Schaid et al. [15]. The benefit of this method is that if the null hypothesis is rejected, showing evidence of true DMRs, the kernel matrix **A** can serve as a smoother, so that smoothed fitted values can be computed and then plotted versus chromosome positions. The peaks in smoothing plot would then be used to detect and locate DMRs.

In order to detect clustering of risk variants for case-control data, Schaid et al. [15] used $Q={\left(O-E\right)}^{\prime }A\left(O-E\right)$ as the kernel distance statistic, where O is the vector of variant counts for cases at different SNPs and E is the vector of expected counts under the null hypothesis, which is estimated from the total counts among cases and controls. The kernel matrix **A** is used to determine how rapid similarity decreases to 0 as the distance between the variants increases, since the association decreases as the distance of two SNPs increases. Schaid et al. [15] suggested using a tri-weight function $A_{jl}={\left(1-{\left(d_{jl}/\tau \right)}^{2}\right)}^{3}$, if $d_{jl}\leq 1$ and 0 otherwise, where $d_{jl}$ is the distance between SNPs *j* and *l*. This function has similar shape as a popular non-compact Gaussian function $A_{ij}=e^{-\frac{d_{ij}^{2}}{\tau }}$ with similar scaled distance. 

SSM was first introduced by [16] to detect clusters in a point process in the one-dimensional setting. With moving windows, the maximum number of points in the windows is recorded and compared to its distribution under the null hypothesis of a purely random Poisson process. A reasonable method that takes into account accurate underlying distributions of methylation counts needs to be developed. Kulldorff et al. [17] proposed a likelihood-based SSM, which was extended to detect genetic variants by [18] by considering the Bernoulli distribution of variants at each position for every individual. The scan statistic is calculated from the likelihood ratio of the frequencies of variants carried among cases and controls within a window versus outside the window, with moving windows along the whole genome. The maximum of the scan statistics over the windows of all possible sizes is defined as the global statistic. However, the approach considered by [18] may not be appropriate for methylation data, since methylated counts at each CpG site for every individual, conditional on the sequencing coverage, follow a binomial distribution instead.

Here, we propose a binomial SSM, which assumes a binomial distribution for the methylation data. Similarly, we also propose a KDM based on [15]. In both approaches, we use logistic regression on methylation rates to adjust for covariates, including sample-specific covariates such as batch effect, in addition to other confounding variables and predictors. 

The details for these statistical methods are presented in Materials and Methods, and the results of our simulation studies are presented in Simulation Results. The methods are applied to a bisulfite- sequenced data from a chronic lymphocytic leukemia (CLL) study [19], with the results presented in Analysis of CLL Data, followed by conclusions and discussions presented in Discussion.

## 2. Materials and Methods

### 2.1. Kernel Distance Method

We modified the KDM, proposed by [15], to model methylation rates, using a tri-weight kernel function to measure the correlation of the methylation rates at different CpG sites as a function of the distance between the sites. This is necessary, since the correlation of methylation rates between CpG sites decreases as the distance between the sites increases.

To facilitate the discussion of the kernel distance method, let $m_{kij}$ be the count of the methylated molecules at CpG site j of individual i in group k, where k=A for cases and U for controls. We assume that $m_{kij}\sim Binom\left(c_{kij},p_{kij}\right)$, where *Binom*() stands for binomial distribution, $c_{kij}$ is a positive integer denoting the coverage, and $p_{kij}$ is the methylation rate at CpG site j for individual i in group k, $k=A,U;\;i=1,2,\ldots ,\;n_{k}$; j=1,2,…,s.

To adjust for confounding factors and linear predictors such as age and gender, we first use logistic regression to fit all data from both groups, using the model,

(1) $$\log\left(\frac{p_{kij}}{1-p_{kij}}\right)=\log\left(\frac{m_{kij}}{c_{kij}-m_{kij}}\right)=\beta_{0}+\beta_{1}x_{ki},$$

where $\beta_{0}$ and $\beta_{1}$ are regression coefficients and $x_{ki}$ represents the vector of covariates of individual i in group k. The fitted odds are calculated for methylation at CpG site j for individual i in group k, to get the corresponding expected methylation rates,

(2) $${\hat{p}}_{kij}=\frac{\exp\left({\hat{\beta }}_{0}+{\hat{\beta }}_{1}x_{ki}\right)}{1+\exp\left({\hat{\beta }}_{0}+{\hat{\beta }}_{1}x_{ki}\right)}.$$

The difference between the observed and expected methylated counts at CpG site j for individual i in group k is calculated as “the adjusted methylation count”,

(3) $$r_{kij}=m_{kij}-{\hat{p}}_{kij}c_{kij}.$$

Define $r_{Aj}=\sum_{i=1}^{n_{A}}r_{Aij}$ and $r_{Uj}=\sum_{i=1}^{n_{U}}r_{Uij}$, then the group effects for cases and controls are quantified as ${\hat{\beta }}_{Aj}=\frac{r_{Aj}}{C_{Aj}}$ and ${\hat{\beta }}_{Uj}=\frac{r_{Uj}}{C_{Uj}}$, where $C_{Aj}=\sum_{i=1}^{n_{A}}c_{Aij}$ and $C_{Uj}=\sum_{i=1}^{n_{U}}c_{Uij}$. The difference between the two groups, $\delta_{j}={\hat{\beta }}_{Aj}-{\hat{\beta }}_{Uj}$, is calculated at each CpG site, and used in the quadratic statistic $Q=\delta^{\prime }A\delta$, where A is a pre-defined matrix of the correlation of methylation rates among CpG sites. 

Generally, the correlation of methylation rates decreases as the distance between the two CpG sites increases. Therefore, the kernel matrix A should be based on a function that determines how rapid the correlation decreases to 0 as the distance increases. We use the tri-weight function $A_{jl}\left(\tau \right)={\left(1-{\left(d_{jl}^{\prime }\left(\tau \right)\right)}^{2}\right)}^{3}$, if $d_{jl}^{\prime }\leq 1$ and 0 otherwise [15], where $d_{jl}^{\prime }\left(\tau \right)=d_{jl}/\tau$ is a scaled distance based on the unknown scaling factor τ, and $d_{jl}$ measures the distance between CpG sites j and l. 

Since the lengths and number of DMRs are unknown and difficult to predict, and the lengths of DMRs vary across the genome, it is difficult to determine the scaling factor that represents the cluster size. When an appropriate size of clusters cannot be predicted and many clusters are expected, it is common to repeat the procedure using different values of τ. Tango [20] suggests allow τ to vary continuously from a small value near zero upwards until τ reaches about half the size of the region of interest. In this manuscript, as proposed by Schaid et al. [15], we consider 10 values of τ, evaluate kernel distance statistic at each value, and select the one that maximizes the statistic; that is,

$$\underset{\tau }{\max}Q=\underset{\tau }{\max}\delta^{\prime }A\left(\tau \right)\delta$$

When a single test statistic is computed based on one scaling factor, the distribution of the kernel distance statistic can be approximated by a scaled chi-square distribution [20]. However, because of multiple scaling factors in our case, scaled chi-square may not be a very good approximation for the distribution of the statistic, and hence we use the permutation method, instead. 

When the null hypothesis is rejected, the scaling factor, $\tau^{*}$, that corresponding to the maximum Q value is accepted as the length of DMR, and the corresponding kernel distance statistic is calculated as,

$$Q\left(\tau^{*}\right)=\overset{m}{\underset{j=1}{\sum }}\overset{m}{\underset{l=1}{\sum }}\left(A_{jl}\left(\tau^{*}\right)\delta_{j}\delta_{l}\right),$$

where m is the number of CpG sites in a genomic region. The percent contribution to $Q\left(\tau^{*}\right)$ at each CpG site is calculated as $U_{j}\left(\tau^{*}\right)/Q\left(\tau^{*}\right)$, where $U_{j}\left(\tau^{*}\right)=\sum_{l=1}^{m}\left(A_{jl}\left(\tau^{*}\right)\delta_{j}\delta_{l}\right)$. The distribution of methylation rates can now be plotted based on the percent contribution $U_{j}\left(\tau^{*}\right)/Q\left(\tau^{*}\right)$ versus CpG site j, which gives a graphical view of potential DMRs. 

### 2.2. Binomial Scan Statistic Method

Scan statistic method can be used as an alternative to KDM for detecting DMRs associated with the disease status. SSM is a likelihood-based approach that uses the likelihood ratio to test whether the methylation rates are different between groups. We use moving windows along the genome, with multiple window sizes, allowing more accurate evaluation of the location and sizes of DMRs. 

Since the methylation rate at each CpG site is correlated with those at the adjacent CpG sites, these correlations are first adjusted by using mixed-effect logistic regression model (see Appendix A for details). Then the “adjusted methylation count” $r_{kij}$ for group *k* is calculated, using Equations (2) and (3). The mixed-effect logistic regression model also allows us to account for relevant covariates. 

We also incorporate an approach proposed by [21] in our proposed SSM to adjust for the clustering structure within each CpG site. By treating the cluster size as random, we can account for the unequal sequencing coverage for individuals at each CpG site. Using the method proposed by [22], the design effect due to clustering is calculated for each CpG site, and used to calculate the adjusted methylation counts ${\tilde{r}}_{kj}$ and sequencing coverage ${\tilde{C}}_{kj}$ (See Appendix A for details). 

We assume that ${\tilde{r}}_{Aj}\sim Binom\;\left({\tilde{C}}_{Aj},\;p_{A}\right)$ and ${\tilde{r}}_{Uj}\sim Binom\;\left({\tilde{C}}_{Uj},\;p_{U}\right)$, where $p_{A}$ and $p_{U}$ are the methylation rates in cases and controls, respectively.

Let $\eta_{k}=\log\left(\frac{p_{k}}{1-p_{k}}\right)$ be the logit transformation of methylation rates of group k within the specific region. In order to test the hypotheses $H_{0}:\eta_{A}=\eta_{U}$ versus $H_{1}:\eta_{A}\neq \eta_{U}$, we propose a test statistic that uses the log of the ratio of the likelihood under $H_{1}$ versus $H_{0}$, which is referred to as the scan statistic. It is given by (see Appendix A for details)

$$\Delta =\frac{T}{\Phi }\left(r_{A}\log\left(\frac{r_{A}}{b_{A}}\right)+\left(\frac{b_{A}}{T}-r_{A}\right)\log\left(1-T\frac{r_{A}}{b_{A}}\right)+\left(1-r_{A}\right)\log\left(\frac{1-r_{A}}{1-b_{A}}\right)+\left(\frac{1-b_{A}}{T}-1+r_{A}\right)\log\left(1-T\frac{1-r_{A}}{1-b_{A}}\right)\;\right)-\frac{1-T}{\Phi }\log\left(1-T\right)$$

and, $b_{A}=\frac{\sum_{j=1}^{s}{\tilde{C}}_{Aj}}{\sum_{j=1}^{s}{\tilde{C}}_{Aj}+\sum_{j=1}^{s}{\tilde{C}}_{Uj}}$, $r_{A}=\frac{\sum_{j=1}^{s}{\tilde{r}}_{Aj}}{\sum_{j=1}^{s}{\tilde{r}}_{Aj}+\sum_{j=1}^{s}{\tilde{r}}_{Uj}}$, $T=\frac{\sum_{j=1}^{s}{\tilde{r}}_{Aj}+\sum_{j=1}^{s}{\tilde{r}}_{Uj}}{\sum_{j=1}^{s}{\tilde{C}}_{Aj}+\sum_{j=1}^{s}{\tilde{C}}_{Uj}}$ and $\Phi =\frac{1}{\sum_{j=1}^{s}{\tilde{C}}_{Aj}+\sum_{j=1}^{s}{\tilde{C}}_{Uj}}$.

One of the advantages of SSM is that the method can easily be extended to more than two groups, if the groups are classified based on nominal responses. Under the multinomial set up, SSM can be used to test the overall hypotheses of no difference in methylation rates among the groups (See Appendix A).

The scan statistic is calculated for each window using moving windows with variable window (VW) size approach across the whole genome. DMR is defined as the window with the highest value of the scan statistic. Thus, for each window W of size w, the binomial scan statistic is be calculated, and the one with highest value denoted by *LR*_w_. Then the maximum of *LR*_w_ over all values of w is used as the global test statistic.

$$i.e.,\;LR=\underset{w}{\max}LR_{w}.$$

The *LR* calculation is unstable if the frequency of methylated counts within a given window is 0 for either cases or controls. To overcome this issue, a pseudo-count of 1 is added to the adjusted methylated and unmethylated counts at each CpG site, these additions implicitly assume that the null hypothesis of no differential methylation is true at all sites. Since the distribution of scan statistic is unknown, an approximate *p*-value for the window with the largest *LR_w_* is calculated using permutation method. 

For case-control studies, SSM is expected to have higher power than the KDM, since SSM using moving window with variable window sizes overcomes the difficult problem of determining the value of scaling factor τ in the KDM. The use of moving windows can also result in more accurate regions of DMRs. 

### 2.3. Simulation

We conducted extensive simulation studies to evaluate the performances of both SSM and KDM. They were compared with respect to the empirical type I error, empirical power and computational efficiency.

Since we used logistic regression for both methods to adjust for covariates, for simplicity, we did not include any covariates in the simulation. Although there are many DMRs along the genome, for the power comparisons for various alternate hypotheses at various significant levels, we assumed that there was only one DMR, so that we only simulated a small genome region around the DMR. We simulated two different scenarios with respect to number of CpG sites in the region, 24 and 30, and all CpG sites within the region were equally spaced.

#### Simulation Parameters

We considered $N_{1}$ cases and $N_{2}$ controls and assumed every individual had equally spaced m CpG sites in the simulated region, of which r consecutive CpG sites in the middle were in the DMR.

Methylation counts at each CpG site for every individual were assumed to be distributed as $B\left(c_{kij},p_{kij}\right)$, k=A,U, i=1,2,…,N, j=1,2,…,m. The sequencing coverage $c_{kij}$ were allowed to vary by sampling the value of it from N(30,13) and then rounding it to the nearest integer, with a minimum of 5 based on the real data analysis by [20]. The correlated methylation rates $p_{kij}$ were simulated using a two-step procedure proposed by [23] in order to model the spatial dependence of the methylation rates among nearby CpG sites. 

First, independent random samples $X_{kij}$ were generated from Beta-distribution for CpG site j of individual i in group k. Under the null hypothesis, $X_{kij}$ were generated as $X_{kij}\sim Beta\left(\alpha_{U},\beta_{U}\right)$, k=A, U. Under the alternative hypothesis, for CpG sites outside the DMR, $X_{kij}$ were generated under the same distribution. Within the DMR under the alternate hypothesis, $X_{kij}$ were generated as $X_{Aij}\sim Beta\left(\alpha_{A},\beta_{A}\right),$ where $\alpha_{A}\neq \alpha_{U}$ or $\beta_{A}\neq \beta_{U}$ for all CpG sites within the DMR, so that the methylation rates were different between cases and controls within DMR. Based on the property of the Beta distribution, with fixed $\alpha_{U}$, $\beta_{A}$ and $\beta_{U}$, only the values of $\alpha_{A}$ were changed, with effect size defined as $d=\frac{\alpha_{A}}{\alpha_{A}+\beta_{A}}-\frac{\alpha_{U}}{\alpha_{U}+\beta_{U}}$. 

For each individual in each group, the vector of independent random variables $X_{ki}$ was transformed into a vector of correlated random variables with correlated methylation rates $p_{ki}=1-\Phi \left(C\Phi^{-1}\left(1-X_{ki}\right)\right)$, where Φ(·) denoted the cumulative distribution function of the standard normal distribution function with Cholesky decomposition C of the correlation matrix $\Sigma =CC^{\prime }$. All diagonal elements of the correlation matrix Σ were 1, and the (i,j)th off-diagonal element was defined as the correlation coefficient ρ divided by the distance between CpG sites i and j, in order to account for the fact that the correlation of methylation rates for two CpG sites decreases as the distance increases. 

## 3. Results

### 3.1. Simulation Results

Simulations were conducted at significance levels of 0.05 and 0.01, total sample sizes of 48 and 60 with equal sample sizes in each group, and regions of 24 and 30 CpG sites with 6 sites in the middle constituting the DMR. We assumed correlation coefficients of ρ=0.7 and ρ=0.5 for methylation rates between adjacent CpG sites, and those among non-adjacent sites were scaled down by dividing ρ by the distances between sites. We set $\alpha_{U}=0.1$, $\beta_{A}=\beta_{U}=0.9$, and used different values of $\alpha_{A}$ to get different effect sizes. Since we simulated DMRs with length of 6 CpG sites, we used τ=6 in KDM, and moving window of size 6 in SSM. 

**Figure 1:**
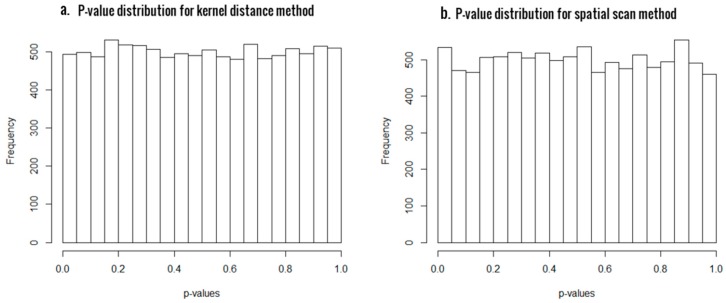
Histogram of *p*-values from scan statistic method (SSM) (panel **a**) and kernel distance statistic (KDM) (panel **b**) for sample size of 48, with 24 CpG sites.

First of all, we generated 10,000 simulated samples using $\alpha_{A}=0.1$ and computed the *p*-values and the empirical type I errors at significant levels 0.05 and 0.01, in order to evaluate the statistical validity of the two approaches. The results are presented in Table 1, and the histogram plots of *p*-values for SSM and KDM in Figure 1a,b, respectively. For a statistical test to be valid, the *p*-values must be uniformly distributed between 0 and 1 under the null hypothesis. As evident from Figure 1, the *p*-value distributions are very close to uniform in both the cases, thus asserting the statistical validity of both our proposed methods. Also, the empirical type I errors are very close to the significant levels, confirming that both methods have excellent control of type I errors. 

**Figure 2:**
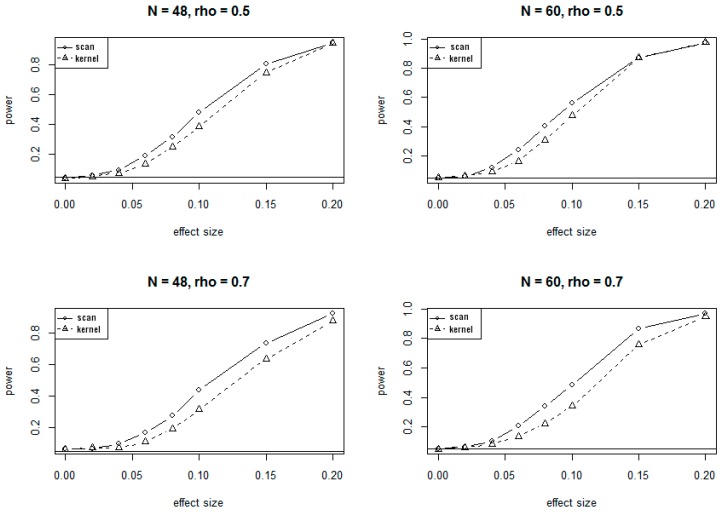
Power curves for SSM and KDM with 24 CpG sites at α=0.05. rho (ρ) is the correlation of methylation rates between adjacent CpG sites.

**Figure 3:**
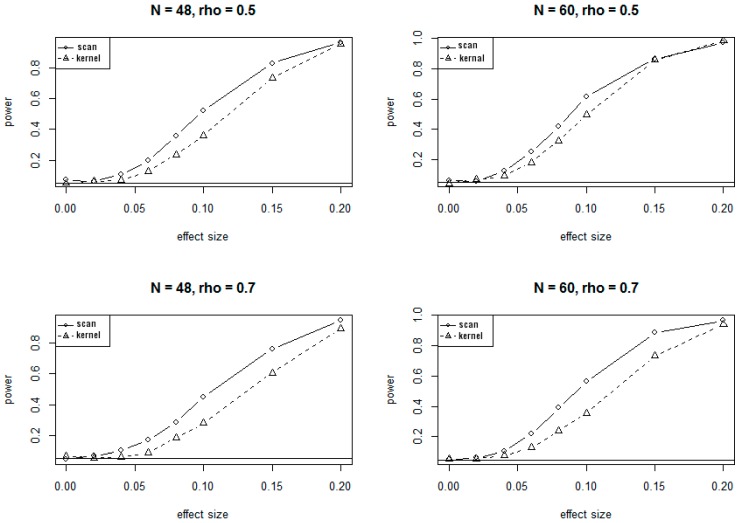
Power curves for SSM and KDM with 30 CpG sites at α=0.05. rho (ρ) is the correlation of methylation rates between adjacent CpG sites.

Because of the massive computing time needed for simulations under the alternate hypotheses, only 1000 simulations were conducted to evaluate the power of SSM and KDM under various alternate scenarios. The plots of power versus different values of effect sizes and correlations at 5% significance level are presented in Figure 2 and Figure 3, corresponding to the 24-site and 30-site regions, respectively. The plots show that values of the power for both SSM and KDM increase as the effect sizes increase, and as well as the sample sizes increase. It is also evident from the plots that SSM has uniformly higher power than KDM.

The conclusions on power at 1% significant level are very similar to and consistent with that at 5% significance level, showing consistently higher power for SSM compared to KDM.

### 3.2. Analysis of Chronic Lymphocytic Leukemia Data

We applied our proposed methods to the methylation data from a genome-wide study of chronic lymphocytic leukemia (CLL), which manifests as a result of clonal expansion of malignant B cells. Research in CLL has identified several molecular alternations that are associated with prognostic values. These include specific cytogenetic patterns [24], mutational status of the immunoglobulin heavy chain variable gene (IgVH) [25] and expression of CD38 [26]. It has been observed that patients with lower levels of CD38 have slower disease progression [25,27].

CD19+ B cells from peripheral blood were collected from 40 subjects [19]. Based on CD38 levels, the samples were categorized as low- vs. high-risk, with 23 samples having CD38 levels ≤20 (low risk) and 17 samples having CD38 levels >20 (high risk). 

Illumina reduced representation bisulfate sequencing [28] was used to generate sequencing reads for each sample, with average sequencing depth per CpG between 32x and 43x, which provided counts of DNA molecules that were methylated and unmethylated at each CpG site [19]. Tango [20] pointed out that aberrant DNA methylation associated with CLL were located more frequently on chromosome 19. So, we analyzed genome-wide methylation data on 17, 917 CpG sites on Chromosome 19 using both SSM and KDM to identify DMRs between high-risk and low-risk CLL subjects.

**Figure 4:**
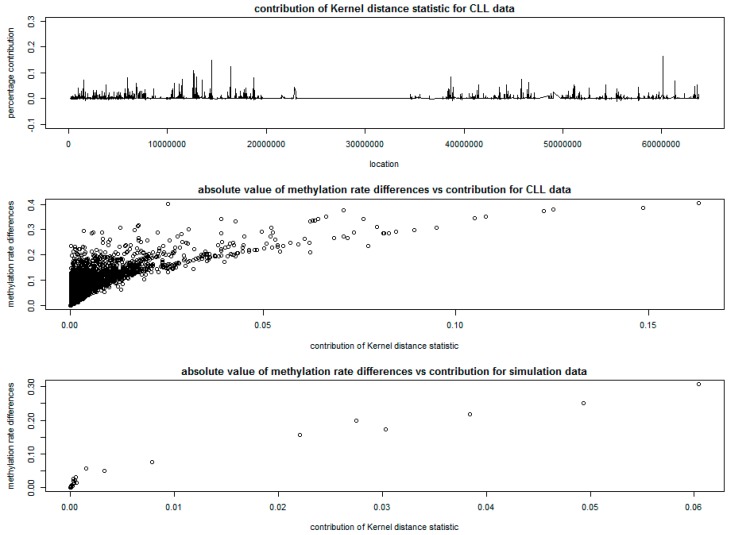
Results of kernel distance method for of chronic lymphocytic leukemia (CLL) data.

The percentage contribution of each CpG site to the kernel distance statistic is plotted at the top of Figure 4. The middle and bottom parts of Figure 4 give the plots of the absolute differences of methylation rates at each CpG site versus the percentage contribution of each CpG site to the kernel distance statistic, based on the CLL data and the simulation data. The absolute value of differences in methylation rates between cases and controls were calculated based on the ratio of adjusted methylation counts and sequencing coverage based on [20] at each CpG site for cases and controls. 

The wedge shapes in both middle and bottom of Figure 4 show that, a large number of CpG sites with small differences in methylation rates have very small contributions to the kernel distance statistic and are possibly not differentially methylated, while the CpG sites with large contributions to the kernel distance statistic show evidence of differential methylation. This indicates the ability of KDM in detecting DMRs, especially using the tri-weight kernel function to incorporate the correlation structure of methylation rates between CpG sites.

The SSM approach detected a total of 66 DMRs with varying window sizes, that containing different number of CpG sites, with a total of 1355 CpG sites (about 7.5% of all CpG sites in Chromosome 19). The top 20 DMRs with highest scan statistic are presented in Table 2, which matches well with the peaks in Figure 4, indicating consistency between SSM and KDM.

The start and end positions of base pairs for each detected DMR were used in the UCSC genome browser (http://genome.ucsc.edu/) to find the genes in the regions. Some of the genes detected in our study include the apolipoprotein gene cluster (*APOC1*, *APOC2*, *APOE*), which are shown to have tight linkage with a chronic lymphocytic leukemia-associated translocation breakpoint [29]. We also detected the genes *CATSPERD*, *PRR22*, *RFX2*, and *MILT1*, which have been shown to be associated with leukemia [30]. For example, translocation and fusion of *MILT1* with myeloid lymphoid leukemia could result in potent oncogenic activity [31,32].

Several studies have suggested that the transcription factor *CREB* (cyclic AMP response element binding protein) may have a role in the pathogenesis of human acute myeloid leukemia (AML) and other cancers [33,34]. In our data, replication factor *C3* is detected whose expression has been reported to have a direct correlation with *CREB* in AML cell lines, as well as in the AML cells from the patients [35]. It is suggested that *C3* may have a role in neoplastic myelopoiesis by promoting the G1/S progression. Another detected gene, *LAIR1*, also has been found to have a correlation with *CREB* [36]. A pathway starts with *LAIR1*, activates downstream *CREB* in AML cells, and sustains the survival and self-renewal of AML stem cells. As a result, inhibition of expression of the immunoreceptor tyrosine-based inhibition motif (ITIM)-containing receptor *LAIR1* does not affect normal hematopoiesis but abolishes leukemia development [36]. 

## 4. Discussion

Results from our simulation studies and the analysis of CLL data indicate that both methods, SSM and KDM, are valid approaches to detect DMRs. Both methods detect DMRs, while allowing for covariates as well as correlation between CpG sites. 

The tri-weight function used in KDM allows for a correlation structure in which the correlation decreases as the distance between CpG sites increases, while SSM use a mixed effects model to incorporate the correlation structure. Although compound symmetry assumption used in SSM may not truly represent actual correlation structure, the sandwich estimates of the fixed effects are appropriate even when the correlation structure is mis-specified, with some trade off of the flexibility for robustness of inference. Our simulation results also show that the mixed effects model is able to adjust for correlation when the simulated correlations decrease as the distances between CpG sites increase. Since the correlation structure can be complicated for methylation data, it may not be easy to find a statistical model that incorporates the correlation structure in its fully complex form.

Both SSM and KDM have reasonable power and good controls of type I error in detecting DMRs between two groups, though SSM performs better in this respect compared to KDM. One reason might be that SSM is a likelihood-based method while KDM is a non-parametric method. Another reason for increased power for SSM could be the use of moving windows with multiple window sizes, which eliminates the difficulty of determining the value of τ in KDM. However, the use of moving windows with a mixed effects model for adjusting the correlation of methylation rates results in substantially longer computation time for SSM. 

In addition, SSM accounts for within cluster correlation by incorporating the method proposed by Xu et al. [20]. SSM also has the advantage that it can be used for comparing methylation rates in more than two groups, while KDM can only be used for comparing two groups. But SSM still has a limitation that it cannot consider the ordering of the group responses because the maximum likelihood estimates are very difficult to obtain when constrained space based on ordering is required.

The uncertainty of τ not only leads to disadvantages in terms of power for KDM, but also it causes KDM to detect only DMRs of approximate lengths, since the kernel distance statistic is calculated using only one value of τ. In reality, the lengths of DMRs range from hundreds of base pair as in small CpG islands, to millions of base pairs in cancer aberrations. It is very difficult to know the exact length of DMRs, a limitation very common in statistical genomics, not only for detecting DMRs but also for detecting rare variants [15]. Use of cross-validation or bootstrapping might help improve the estimation of the window sizes. 

Another reason for the lower power for KDM compared to SSM may be that KDM is not able to adjust for unequal sequencing coverage for all individuals at each CpG site, while SSM incorporates the method proposed by Xu et al. [20] to adjust sequencing coverage and methylation counts. One possible solution is to use a mixed-effect logistic model with random intercept to adjust for the within cluster correlation, treating methylation data at each CpG site as a cluster. 

We have only focused on DNA methylation data in developing both our methods. However, large-scale cancer genomics projects such as TCGA (The Cancer Genome Atlas Research Network) are currently generating multiple layers of genomics data for early tumor, including DNA copy number, methylation, and mRNA expression. Statistical methods for integrated analyses and systematic modeling of such genomics data deserve more attention.

## Figures and Tables

**Table 1:** Type I errors for both kernel distance method (KDM) and scan statistic method (SSM) based on 10,000 simulations.

Significance Level				0.05		0.01	
Total Sample Sizes	Total Number of Sites	$\alpha_{A}$	ρ	KDM	SSM	KDM	SSM
48	24	0.1	0.5	0.053	0.056	0.013	0.014
48	24	0.1	0.7	0.0514	0.0518	0.0116	0.0125

**Table 2:** Results of SSM for CLL data on Chromosome 19; (Start: the starting nucleotide position of the SSM; End: the ending nucleotide position of the SSM).

Start	End	Window Size	*p*-Value	Start	End	Window Size	*p*-Value
951,756	960,480	15	0.001	40,495,154	40,706,271	40	0.033
5,748,848	5,855,704	35	0.024	40,958,295	40,995,281	15	0.028
5,949,493	6,059,920	15	0.037	41,323,151	41,345,137	5	0.027
6,222,967	6,325,326	40	0.042	42,400,872	42,516,823	25	0.023
6,695,897	6,704,448	5	0.039	42,631,539	42,651,999	10	0.022
7,049,880	7,149,391	20	0.042	43,411,447	43,472,750	70	0.023
8,306,311	8,416,558	105	0.02	44,099,619	44,158,078	10	0.003
10,078,223	10,091,192	15	0.049	45,388,832	45,464,209	30	0.007
10,261,108	10,336,402	75	0.048	45,812,107	45,821,840	5	0.005
10,366,854	10,374,990	5	0.011	46,555,659	46,595,121	5	0.007
10,529,295	10,537,824	5	0.033	47,040,515	47,078,316	5	0.008
11,311,211	11,369,166	35	0.046	50,778,928	50,793,474	5	0.021
11,852,835	11,937,174	15	0.012	51,010,992	51,058,089	10	0.029
12,036,638	12,128,243	10	0.019	51,059,619	51,079,866	15	0.026
13,780,707	13,818,691	30	0.035	51,409,109	51,427,742	5	0.027
15,871,811	15,874,720	5	0.033	53,821,358	53,829,676	15	0.001
16,211,533	16,298,141	10	0.008	53,914,126	53,934,314	20	0.011
16,779,596	16,818,698	5	0.049	53,946,213	53,983,289	5	0.033
17,181,376	17,207,209	20	0.032	54,819,984	54,835,037	5	0.035
17,483,944	17,492,848	5	0.027	54,872,826	54,884,388	10	0.033
18,358,107	18,358,200	5	0.018	55,714,472	55,760,862	15	0.047
18,839,769	18,849,925	40	0.037	55,853,400	55,911,789	30	0.046
19,196,863	19,220,558	10	0.001	56,884,684	56,887,726	5	0.002
20,751,241	20,751,405	10	0.016	58,388,434	58,388,478	5	0.006
21,443,528	21,449,542	5	0.042	58,980,127	59,064,230	15	0.007
35,558,112	35,558,143	5	0.014	59,643,525	59,652,071	5	0.002
37,528,315	37,528,707	10	0.035	59,652,664	59,666,539	15	0.011
37,808,618	37,858,100	10	0.019	60,109,922	60,545,979	205	0.04
38,315,030	38,359,639	10	0.012	60,790,219	60,808,074	15	0.015
38,576,223	38,632,218	20	0.001	61,304,533	61,424,810	55	0.013
38,980,210	39,003,767	20	0.043	61,741,700	61,798,595	20	0.048
39,760,398	39,760,441	5	0.022	62,277,420	62,310,019	5	0.019
40,193,224	40,214,045	25	0.046	63,565,854	63,570,870	10	0.035

## Appendix A

### A.1. Adjusting for Correlation Between CpG sites with Mixed-Effects Model

A random slope and random intercept logistic regression model is considered for modeling methylation counts at each CpG site for every individual, which is given by

(A1) $$\log\left(\frac{p_{kij}}{1-p_{kij}}\right)=\log\left(\frac{m_{kij}}{c_{kij}-m_{kij}}\right)=\beta_{0}+\beta_{1}s_{j}+\beta_{2}x_{ki}+\nu_{0ki}+\nu_{1ki}s_{j},$$

where $x_{ki}$ is the covariate, and $s_{j}$ represents the distance of CpG site j from the starting point. The random effect $\nu_{ki}=\left(\begin{array}{c} \nu_{0ki}\\ \nu_{1ki} \end{array}\right)$ is assumed to vary independently across individuals with $\nu_{ki}\sim N\left(0,\left(\begin{array}{cc} \sigma_{\nu_{0ki}}^{2} & \sigma_{\nu_{0ki}}\sigma_{\nu_{1ki}}\\ \sigma_{\nu_{0ki}}\sigma_{\nu_{1ki}} & \sigma_{\nu_{1ki}}^{2} \end{array}\right)\right)$, where $\sigma_{\nu_{0ki}}^{2}$ and $\sigma_{\nu_{1ki}}^{2}$ are the variances of $\nu_{0ki}$ and $\nu_{1ki}$, respectively, and $\sigma_{\nu_{0ki}}\sigma_{\nu_{1ki}}$ is the covariance of $\nu_{0ki}$ and $\nu_{1ki}.$

### A.2. Adjusting for Clustering Structure Within Each CpG Site

Within each CpG site, the design effect due to clustering is calculated using the method proposed by Rao and Scott (1986). To calculate the design effect, we first calculate the adjusted methylation counts $r_{Aj}$ and $r_{Uj}$ at CpG site j in groups *A* (cases) and *U* (controls), respectively, ignoring the clustering within individuals. That is, $r_{Aj}=\sum_{i=1}^{n_{A}}r_{Aij}$ and $r_{Uj}=\sum_{i=1}^{n_{U}}r_{Uij}$. Then the group effects are estimated as, ${\hat{\beta }}_{Aj}=\frac{r_{Aj}}{C_{Aj}}$ and ${\hat{\beta }}_{Uj}=\frac{r_{Uj}}{C_{Uj}}$, where $C_{Aj}=\sum_{i=1}^{n_{A}}c_{Aij}$ and $C_{Uj}=\sum_{i=1}^{n_{U}}c_{Uij}$. The variances of the group effects are given by

$$\hat{V}\left({\hat{\beta }}_{Aj}\right)=\frac{n_{A}\sum_{i=1}^{n_{A}}{\left(r_{Aij}-c_{Aij}{\hat{\beta }}_{Aj}\right)}^{2}}{(n_{A}-1)C_{Aj}^{2}}\;\mathrm{and}\;\hat{V}\left({\hat{\beta }}_{Uj}\right)=\frac{n_{U}\sum_{i=1}^{n_{U}}{\left(r_{Uij}-c_{Uij}{\hat{\beta }}_{Uj}\right)}^{2}}{(n_{U}-1)C_{Uj}^{2}}.$$

Without clustering, the variances of the group effects under the binomial distribution are

$${\hat{V}}_{B}\left({\hat{\beta }}_{Aj}\right)=\frac{{\hat{\beta }}_{Aj}\left(1-{\hat{\beta }}_{Aj}\right)}{C_{Aj}}\;\mathrm{and}\;{\hat{V}}_{B}\left({\hat{\beta }}_{Uj}\right)=\frac{{\hat{\beta }}_{Uj}\left(1-{\hat{\beta }}_{Uj}\right)}{C_{Uj}}.$$

Then the design effects for the two groups are defined as,

(A2) $$d_{Aj}=\frac{\hat{V}\left({\hat{\beta }}_{Aj}\right)}{{\hat{V}}_{B}\left({\hat{\beta }}_{Aj}\right)}\;\mathrm{and}\;d_{Uj}=\frac{\hat{V}\left({\hat{\beta }}_{Uj}\right)}{{\hat{V}}_{B}\left({\hat{\beta }}_{Uj}\right)}.$$

The design effects are then used to calculate the adjusted methylation counts and sequencing coverage at each CpG site in cases and controls as

(A3) $${\tilde{r}}_{Aj}=\frac{r_{Aj}}{d_{Aj}}\;\mathrm{and}\;{\tilde{r}}_{Uj}=\frac{r_{Uj}}{d_{Uj}}$$

(A4) $${\tilde{C}}_{Aj}=\frac{C_{Aj}}{d_{Aj}}\;\mathrm{and}\;{\tilde{C}}_{Uj}=\frac{C_{Uj}}{d_{Uj}}$$

### A.3. Binomial Scan Statistic for Case-Control Studies

Considering ${\tilde{r}}_{kj}\sim Binom\left({\tilde{C}}_{kj},\;p_{k}\right),$ where $p_{k}$ is the methylation rate in group *k*, then the likelihood of ${\tilde{r}}_{kj}$, *k* = *A*, *U*, is given by,

$$\begin{array}{l} f\left({\tilde{r}}_{kj}\right)=\left(\begin{array}{c} {\tilde{C}}_{kj}\\ {\tilde{r}}_{kj} \end{array}\right)p_{k}^{{\tilde{r}}_{kj}}{\left(1-p_{k}\right)}^{{\tilde{C}}_{kj}-{\tilde{r}}_{kj}}\\ =\left(\begin{array}{c} {\tilde{C}}_{kj}\\ {\tilde{r}}_{kj} \end{array}\right)\;exp\left({\tilde{r}}_{kj}\log\left(\frac{p_{k}}{1-p_{k}}\right)+{\tilde{C}}_{kj}\log\left(1-p_{k}\right)\right). \end{array}$$

Since $\left({\tilde{r}}_{k1},\;{\tilde{r}}_{k2},\;\ldots ,\;{\tilde{r}}_{ks}\right)$ for the s consecutive CpG sites are assumed to be independent, the joint likelihood of adjusted methylation counts over s consecutive CpG sites in the defined region for group k is the product of the likelihoods of the s CpG sites, which can be expressed as,

$$\begin{array}{r} f\left({\tilde{r}}_{k1},\;{\tilde{r}}_{k2},\;\ldots ,\;{\tilde{r}}_{ks}\right)=\overset{s}{\underset{j=1}{\prod }}\left(\begin{array}{c} {\tilde{C}}_{kj}\\ {\tilde{r}}_{kj} \end{array}\right)exp\left({\tilde{r}}_{kj}\log\left(\frac{p_{k}}{1-p_{k}}\right)+{\tilde{C}}_{kj}\log\left(1-p_{k}\right)\right)\;\;\\ =\overset{s}{\underset{j=1}{\prod }}\left(\begin{array}{c} {\tilde{C}}_{kj}\\ {\tilde{r}}_{kj} \end{array}\right)exp\left\{\overset{s}{\underset{j=1}{\sum }}{\tilde{C}}_{kj}\left(\frac{\sum_{j=1}^{s}{\tilde{r}}_{kj}}{\sum_{j=1}^{s}{\tilde{C}}_{kj}}\log\left(\frac{p_{k}}{1-p_{k}}\right)+\log\left(1-p_{k}\right)\right)\right\}. \end{array}$$

From this likelihood, we can see the distribution of adjusted methylated counts follow a one-parameter exponential family $y=\left({\tilde{r}}_{k1},\;{\tilde{r}}_{k2},\;\ldots ,\;{\tilde{r}}_{ks}\right)\sim EXP\left(\eta ,\phi ,T,B_{e},a\right)$ with

$$T\left({\tilde{r}}_{k1},\;{\tilde{r}}_{k2},\;\ldots ,\;{\tilde{r}}_{ks}\right)=\frac{\sum_{j=1}^{s}{\tilde{r}}_{kj}}{\sum_{j=1}^{s}{\tilde{C}}_{kj}}$$

$$\eta =log\left(\frac{p_{k}}{1-p_{k}}\right)\;where\;p_{k}=\frac{\exp\left(\eta \right)}{1+\exp\left(\eta \right)}$$

$$B_{e}\left(\eta \right)=-\log\left(1-p_{k}\right)=\log\left(1+e^{\eta }\right)$$

$$\phi =\frac{1}{\sum_{j=1}^{s}{\tilde{C}}_{kj}}$$

a(ϕ)=1

and the log-likelihood $l\left(\eta ;y\right)=\left(\eta T\left(y\right)-B_{e}\left(\eta \right)\right)/\phi$ after ignoring an additive constant that does not depend on η. Based on this likelihood function, we can find the maximum likelihood estimator (MLE) of parameter η in $EXP\left(\eta ,\phi_{i},T,B_{e},a\right)$ as $\hat{\eta }=g_{e}\left(T\left(y\right)\right)$, where $g_{e}={\left(B_{e}^{\prime }\right)}^{-1}=\log\left(T\right)-\log\left(1-T\right)$ [37].

Then the scan statistic as the ratio of the likelihood under $H_{1}$ versus $H_{0}$, given by

(A5) $$\Delta =\kappa \left(T_{A},\;\Phi_{A}\right)+\kappa \left(T_{U},\Phi_{U}\right)-\kappa \left(T,\Phi \right),$$

where $\kappa \left(x,y\right)=\left(xg_{e}\left(x\right)-B_{e}\left(g_{e}\left(x\right)\right)\right)/y$, $\frac{1}{\Phi }=\frac{1}{\Phi_{A}}+\frac{1}{\Phi_{U}}$, $T=b_{A}T_{A}+\left(1-b_{A}\right)T_{U}$, and $b_{A}=\frac{1}{\Phi_{A}}/\left(\frac{1}{\Phi_{A}}+\frac{1}{\Phi_{U}}\right)$.

Here we have, $\Phi_{A}=\frac{1}{\sum_{j=1}^{s}{\tilde{C}}_{Aj}}$, $\Phi_{U}=\frac{1}{\sum_{j=1}^{s}{\tilde{C}}_{Uj}}$, $T_{A}=\frac{\sum_{j=1}^{s}{\tilde{r}}_{Aj}}{\sum_{j=1}^{s}{\tilde{C}}_{Aj}}$, and $T_{U}=\frac{\sum_{j=1}^{s}{\tilde{r}}_{Uj}}{\sum_{j=1}^{s}{\tilde{C}}_{Uj}}$ for cases and controls, with

$$\Delta =\frac{T}{\Phi }\left(r_{A}\log\left(\frac{r_{A}}{b_{A}}\right)+\left(\frac{b_{A}}{T}-r_{A}\right)\log\left(1-T\frac{r_{A}}{b_{A}}\right)+\left(1-r_{A}\right)\log\left(\frac{1-r_{A}}{1-b_{A}}\right)+\left(\frac{1-b_{A}}{T}-1+r_{A}\right)\log\left(1-T\frac{1-r_{A}}{1-b_{A}}\right)\;\right)-\frac{1-T}{\Phi }\log\left(1-T\right)$$

and, $b_{A}=\frac{\sum_{j=1}^{s}{\tilde{C}}_{Aj}}{\sum_{j=1}^{s}{\tilde{C}}_{Aj}+\sum_{j=1}^{s}{\tilde{C}}_{Uj}}$, $r_{A}=\frac{\sum_{j=1}^{s}{\tilde{r}}_{Aj}}{\sum_{j=1}^{s}{\tilde{r}}_{Aj}+\sum_{j=1}^{s}{\tilde{r}}_{Uj}}$, $T=\frac{\sum_{j=1}^{s}{\tilde{r}}_{Aj}+\sum_{j=1}^{s}{\tilde{r}}_{Uj}}{\sum_{j=1}^{s}{\tilde{C}}_{Aj}+\sum_{j=1}^{s}{\tilde{C}}_{Uj}}$ and $\Phi =\frac{1}{\sum_{j=1}^{s}{\tilde{C}}_{Aj}+\sum_{j=1}^{s}{\tilde{C}}_{Uj}}$.

### A.4. SSM for Multinomial Responses

Before testing the differences among groups, the methylation counts and sequencing coverage need to be adjusted. First, we use the mixed-effect logistic regression model (A1) to adjust for covariates and the correlation of methylation rates between CpG sites. Then design effects in (A2) are calculated based on Xu et al. [20], and used to adjust the residual ${\tilde{r}}_{kj}$ and the sequencing coverage ${\tilde{C}}_{kj}$ for group k at CpG site j, for all k and j, as in (A3) and (A4).

Assume that all CpG sites in a DMR for group k have same methylation rate $p_{k}$ with adjusted methylation count ${\tilde{r}}_{kj}\sim B\left({\tilde{C}}_{kj},p_{k}\right)$. Let $\eta_{k}=\log\left(\frac{p_{k}}{1-p_{k}}\right)$ be the logit transformation of methylation rates of group k. The hypothesis of interest is,

$$H_{0}:\eta_{1}=\eta_{2}=\ldots =\eta_{K}=\eta \;\mathrm{vs}.\;H_{1}:\;\eta_{1},\eta_{2},\ldots ,\eta_{K}\;\mathrm{not}\;\mathrm{all}\;\mathrm{are}\;\mathrm{equal}.\;$$

Here the groups are assumed to be independent, and the log of the ratio of the likelihood under $H_{1}$ versus $H_{0}$ is used as the test statistic as before, given by,

$$\Delta =\overset{K}{\underset{k=1}{\sum }}\kappa \left(T_{k},\Phi_{k}\right)-\kappa \left(T,\Phi \right),$$

where $\kappa \left(x,y\right)=\left(xg_{e}\left(x\right)-B_{e}\left(g_{e}\left(x\right)\right)\right)/y$ and $T_{k}=\frac{\sum_{j=1}^{s}{\tilde{r}}_{kj}}{\sum_{j=1}^{s}{\tilde{C}}_{kj}}$, $\Phi_{k}=\frac{1}{\sum_{j=1}^{s}{\tilde{C}}_{kj}}$.

Define $\Phi =\frac{1}{\sum_{k=1}^{K}\sum_{j=1}^{s}{\tilde{C}}_{kj}}$ and $T=\frac{\sum_{k=1}^{K}\sum_{j=1}^{s}{\tilde{r}}_{kj}}{\sum_{k=1}^{K}\sum_{j=1}^{s}{\tilde{C}}_{kj}}$, thus $\frac{1}{\Phi }=\sum_{k=1}^{K}\frac{1}{\Phi_{k}}$, $T=\sum_{k=1}^{K}b_{k}T_{k}$, where $b_{k}=\frac{\frac{1}{\Phi_{k}}}{\frac{1}{\Phi }}=\frac{\sum_{j=1}^{s}{\tilde{C}}_{kj}}{\sum_{k=1}^{K}\sum_{j=1}^{s}{\tilde{C}}_{kj}}$. Then the scan statistic for more than two groups is given by,

$$\Delta =\overset{K}{\underset{k=1}{\sum }}\kappa \left(T_{k},{\;\Phi }_{k}\right)-\kappa \left(T,\Phi \right)\;=\overset{K}{\underset{k=1}{\sum }}\frac{T}{\Phi }\left(r_{k}\log\left(\frac{r_{k}}{b_{k}}\right)+\left(\frac{b_{k}}{T}-r_{k}\right)\log\left(1-T\frac{r_{k}}{b_{k}}\right)\;\right)-\frac{1-T}{\Phi }\log\left(1-T\right)$$

where $b_{k}=\frac{\sum_{j=1}^{s}{\tilde{C}}_{kj}}{\sum_{k=1}^{K}\sum_{j=1}^{s}{\tilde{C}}_{kj}}$, $r_{k}=\frac{\sum_{j=1}^{s}{\tilde{r}}_{kj}}{\sum_{k=1}^{K}\sum_{j=1}^{s}{\tilde{r}}_{kj}}$, $\Phi =\frac{1}{\sum_{k=1}^{K}\sum_{j=1}^{s}{\tilde{C}}_{kj}}$, and $T=\frac{\sum_{k=1}^{K}\sum_{j=1}^{s}{\tilde{r}}_{kj}}{\sum_{k=1}^{K}\sum_{j=1}^{s}{\tilde{C}}_{kj}}$.
